# Spontaneous coronary artery dissection in a patient with cerebral autosomal dominant arteriopathy with subcortical infarcts and leucoencephalopathy syndrome: a case report

**DOI:** 10.1093/ehjcr/ytz136

**Published:** 2019-08-26

**Authors:** Nikolaos Tsanaxidis, Sally Elshafie, Shahzad Munir

**Affiliations:** Cardiology Department, Wolverhampton Heart and Lung Centre, New Cross Hospital, Wolverhampton Road, Wolverhampton WV10 0QP, UK

**Keywords:** CADASIL syndrome, Spontaneous coronary artery dissection, Case report

## Abstract

**Background:**

Cerebral autosomal dominant arteriopathy with subcortical infarcts and leucoencephalopathy (CADASIL) syndrome is a genetically inherited condition most notably affecting the central nervous system in young adults. There is limited knowledge on its association with coronary arteries, and its association with spontaneous coronary artery dissection (SCAD) has not been previously reported.

**Case summary:**

A 61-year-old woman who is known to have CADASIL syndrome presented with anterior ST-segment myocardial infarction and underwent emergency angiography. This showed appearance consistent with SCAD in the mid left anterior descending artery with tubular stenosis.

**Discussion:**

The association between CADASIL syndrome and SCAD has not been previously reported. The similarity in the underlying pathophysiology of these two conditions makes this case intriguing.

**Learning points**

- Spontaneous coronary artery dissection could be associated with cerebral autosomal dominant arteriopathy with subcortical infarcts and leucoencephalopathy (CADASIL) syndrome.
- Spontaneous coronary artery dissection is a result of arteriopathy and CADASIL syndrome is a result of angiopathy both similarly affecting the vascular system causing ischaemia and infarction.

## Introduction

Cerebral autosomal dominant arteriopathy with subcortical infarcts and leucoencephalopathy (CADASIL) syndrome is an autosomal dominant inherited angiopathy thought to be caused by mutations in the *NOTCH3* gene.^1^ The NOTCH3 gene encodes for the NOTCH3 receptor protein, modulating vascular smooth muscle activity. Mutations in the gene are thought to result in loss of vascular smooth muscle cells, vascular dysfunction, and the associated subsequent vascular ischaemic events. Its prevalence is estimated as between 2 and 5 in 100 000.^2^ Migraine with aura and cerebral infarctions, with later progressive features of leucoencephalopathy is the most recognized form of presentation.^3^ Histological examination shows granular osmiophilic material (GOM) in arterial walls.^4^ Magnetic resonance imaging shows white matter abnormalities, lacunar infarcts, and micro-bleeds.^5^

We present a case of a patient with CADASIL syndrome who presented with an acute coronary syndrome; subsequent coronary angiography was highly suggestive of spontaneous coronary dissection (SCAD). Spontaneous coronary artery dissection is characterized by a false lumen forming within the layers of the coronary arterial wall restricting blood flow. The underlying mechanism for SCAD is unclear but it is regarded as a manifestation of a more widespread arteriopathy resulting in coronary arterial wall degradation with increased risk of development of compressive intramural haematoma due to vaso vasorum bleeding or frank intimal tear and subsequent ischaemic coronary events.^6^

## Timeline

**Table:** 

Time	Events
5 October 2018	Emergency admission for ST-elevation myocardial infarction. Angiogram showed spontaneous coronary dissection.
6 October 2018	Started on IV glyceryl trinitrate (GTN) infusion in view of chest pain.
7 October 2018	GTN infusion stopped.
9 October 2018	Discharged with dual antiplatelets for 3 months and Aspirin lifelong thereafter. Awaiting follow up in clinic.

## Case presentation

**Figure 1:**
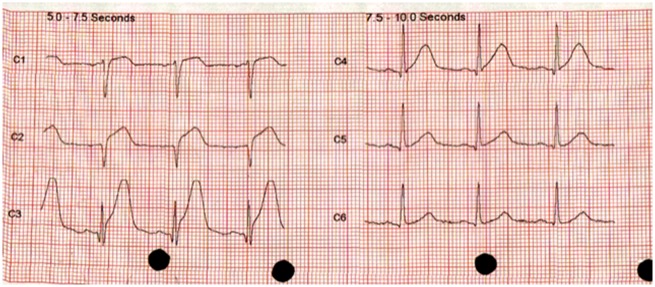
Electrocardiogram on admission.

**Figure 2:**
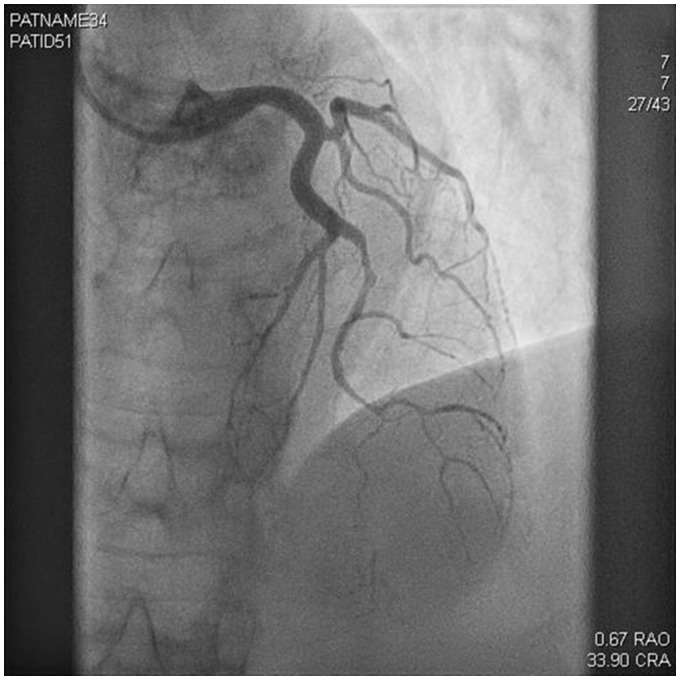
Angiogram of the left anterior descending artery showing spontaneous coronary artery dissection in the mid left anterior descending artery.

**Figure 3:**
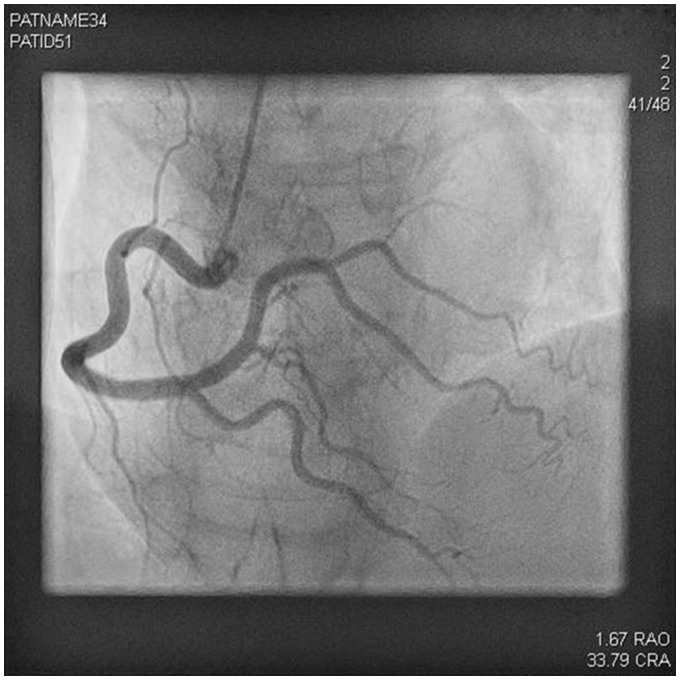
Angiogram showing a normal right coronary artery.

A 61-year-old woman who was normally fit and well with a background history of CADASIL syndrome presented to the emergency department with chest pain and feeling dizzy and unwell. She had no other risk factors for coronary artery disease. CADASIL diagnosis was made in a regional specialist neurological centre and she was under follow-up. Her cardiovascular examination revealed an initial blood pressure of 167/81 mmHg, heart rate of 81 b.p.m., and her electrocardiogram showed anterior ST-segment elevation (*Figure 1*) with a raised initial high-sensitivity troponin level which was 1551 ng/L (0–15 ng/L). On auscultation, her chest was clear and heart sounds were essentially normal. Jugular venous pressure was normal and there was no peripheral oedema present. Her bedside focused transthoracic echocardiography showed anterolateral regional wall motion abnormalities with akinesia in the anterior segments with a visually estimated left ventricular ejection fraction of 35%. She underwent emergency radial coronary angiography which showed an abnormal appearance of the left anterior descending artery (LAD) suggestive of SCAD. She had a long segment of irregular filling of the mid anterior descending artery with a tubular stenosis. The proximal LAD, the circumflex, and right coronary arteries were angiographically normal. Given the appearance of thrombolysis in myocardial infarction Grade 3 flow in the LAD territory and recognizing the potential risks of percutaneous intervention in the setting of SCAD, she was, therefore, managed medically with dual antiplatelets (Aspirin and Clopidogrel) for 3 months, Bisoprolol, Ramipril, and Atorvastatin (*Figures 2* and *3*). The duration of dual-antiplatelet therapy was limited as no stent was deployed and a reported natural history of vascular healing in SCAD. Intravascular imaging was not used to confirm the suspected angiographic diagnosis given the reported vascular complications with instrumentations in SCAD such as extending the coronary dissection with wire or imaging catheter and guide catheter iatrogenic dissection.^7^^,^^8^ A repeat transthoracic echocardiogram has not been performed yet.

## Discussion

Progressive arterial fibrosis and loss of vascular smooth muscle cells, together with deposition of GOM, is the proposed mechanism for the neurological vascular events seen with CADASIL. This pathophysiological phenomenon is seldom described outside the central nervous system, and its association with coronary arteries remains unknown. Coronary arterial changes of a similar pathophysiological nature would explain the potential risk of intramural haematoma or coronary artery dissection developing and the associated myocardial infarctions seen. This would be in the absence of traditional cardiovascular risk factors.^9^^,^^10^

## Conclusion

Here, we describe a case of likely intravascular haematoma and SCAD in a patient with CADASIL syndrome. CADASIL syndrome is an angiopathy associated with predominately central nervous system vascular events. Spontaneous coronary artery dissection as discussed is also regarded as a manifestation of an underlying arteriopathy. We present a case of a patient with CADASIL experiencing SCAD. To our knowledge, an association between the two has not been described, but given the similar underlying pathophysiological mechanisms, further investigation could be warranted. Further invasive angiography was not performed.

## Lead author biography

**Figure:**
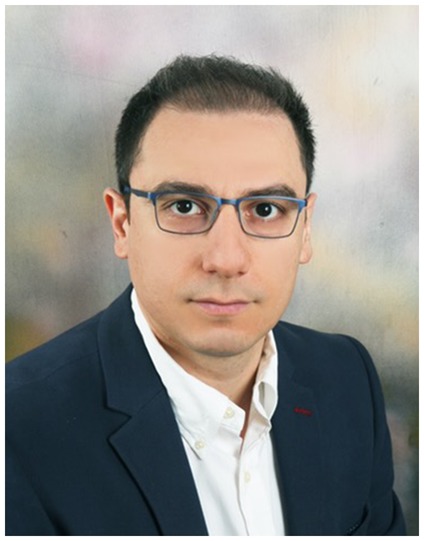


Nikolaos Tsanaxidis is a Senior Interventional Fellow in Cardiology at New Cross Hospital, Heart & Lung tertiary Centre, Royal Wolverhampton NHS Trust, UK. He is a Board Certified Cardiologist in UK and Greece since 2018; also PhD candidate in Larissa University Hospital, Greece. He is a Professional member of European Society of Cardiology (ESC) and EAPCI and member of British Cardiovascular Intervention Society (BCIS).

## Supplementary material

Supplementary material is available at *European Heart Journal - Case Reports* online.

**Slide sets:** A fully edited slide set detailing this case and suitable for local presentation is available online as Supplementary data.

**Consent:** The author/s confirm that written consent for submission and publication of this case report including image(s) and associated text has been obtained from the patient in line with COPE guidance.

**Conflict of interest:** none declared.

## Supplementary Material

ytz136_Supplementary_Slide_Set

## References

[1] Kalimo H, Ruchoux M, Viitanen M, Kalaria R. CADASIL: a common form of hereditary arteriopathy causing brain infarcts and dementia. Brain Pathol 2006;12:371–384. 10.1111/j.1750-3639.2002.tb00451.x PMC8096024 12146805

[2] Di Donato I, Bianchi S, De Stefano N, Dichgans M, Dotti MT, Duering M, Jouvent E, Korczyn AD, Lesnik-Oberstein SAJ, Malandrini A, Markus HS, Pantoni L, Penco S, Rufa A, Sinanović O, Stojanov D, Federico A. Cerebral Autosomal Dominant Arteriopathy with Subcortical Infarcts and Leukoencephalopathy (CADASIL) as a model of small vessel disease: update on clinical, diagnostic, and management aspects. BMC Med 2017;15:1–12. 28231783 10.1186/s12916-017-0778-8 PMC5324276

[3] Chabriat H, Joutel A, Dichgans M, Tournier-Lasserve E, Bousser M. CADASIL. Lancet Neurol 2009;8:643–653. 19539236 10.1016/S1474-4422(09)70127-9

[4] Joutel A, Corpechot C, Ducros A, Vahedi K, Chabriat H, Mouton P, Alamowitch S, Domenga V, Cécillion M, Marechal E, Maciazek J, Vayssiere C, Cruaud C, Cabanis EA, Ruchoux MM, Weissenbach J, Bach JF, Bousser MG, Tournier-Lasserve E. Notch3 mutations in CADASIL, a hereditary adult-onset condition causing stroke and dementia. Nature 1996;383:707–710. 8878478 10.1038/383707a0

[5] Rubin C, Hahn V, Kobayashi T, Litwack A. A report of accelerated coronary artery disease associated with cerebral autosomal dominant arteriopathy with subcortical infarcts and leukoencephalopathy. Case Rep Cardiol 2015;2015:167513. 26435852 10.1155/2015/167513 PMC4575993

[6] Al-Hussaini A, Adlam D. Spontaneous coronary artery dissection. Heart 2017;103:1043–1051. 28363899 10.1136/heartjnl-2016-310320

[7] Rogers JH, Lasala JM. Coronary artery dissection and perforation complicating percutaneous coronary intervention. J Invasive Cardiol 2004;16:493–499. 15353832

[8] Hayes SN, Kim ESH, Saw J, Adlam D, Arslanian-Engoren C, Economy KE, Ganesh SK, Gulati R, Lindsay ME, Mieres JH, Naderi S, Shah S, Thaler DE, Tweet MS, Wood MJ;American Heart Association Council on Peripheral Vascular Disease; Council on Clinical Cardiology; Council on Cardiovascular and Stroke Nursing; Council on Genomic and Precision Medicine; and Stroke Council. Spontaneous coronary artery dissection: current state of the science: a scientific statement from the American Heart Association. Circulation 2018;137:e523–e557. 29472380 10.1161/CIR.0000000000000564 PMC5957087

[9] Langer C, Adukauskaite A, Plank F, Feuchtner G,, Cartes-Zumelzu F. Cerebral Autosomal Dominant Arteriopathy (CADASIL) with cardiac involvement (ANOCA) and subcortical leukencephalopathy. J Cardiovasc Comput Tomogr 2018;doi:10.1016/j.jcct.2018.08.005. 10.1016/j.jcct.2018.08.005 30197288

[10] Lesnik Oberstein SAJ, Jukema JW, Van Duinen SG, Macfarlane PW, van Houwelingen HC, Breuning MH, Ferrari MD, Haan J. Myocardial infarction in cerebral autosomal dominant arteriopathy with subcortical infarcts and leukoencephalopathy (CADASIL). Medicine (Baltimore) 2003;82:251–256. 12861102 10.1097/01.md.0000085054.63483.40

