# Supplementary material for: Spontaneous coronary artery dissection in a patient with cerebral autosomal dominant arteriopathy with subcortical infarcts and leucoencephalopathy syndrome: a case report
Source: Eur Heart J Case Rep. 2019 Aug 26;3(3):ytz136. doi: 10.1093/ehjcr/ytz136 (PMC6764544; doi:10.1093/ehjcr/ytz136)
Supplement: ytz136_Supplementary_Slide_Set [file ytz136_supplementary_slide_set.pptx]

## Slide 1
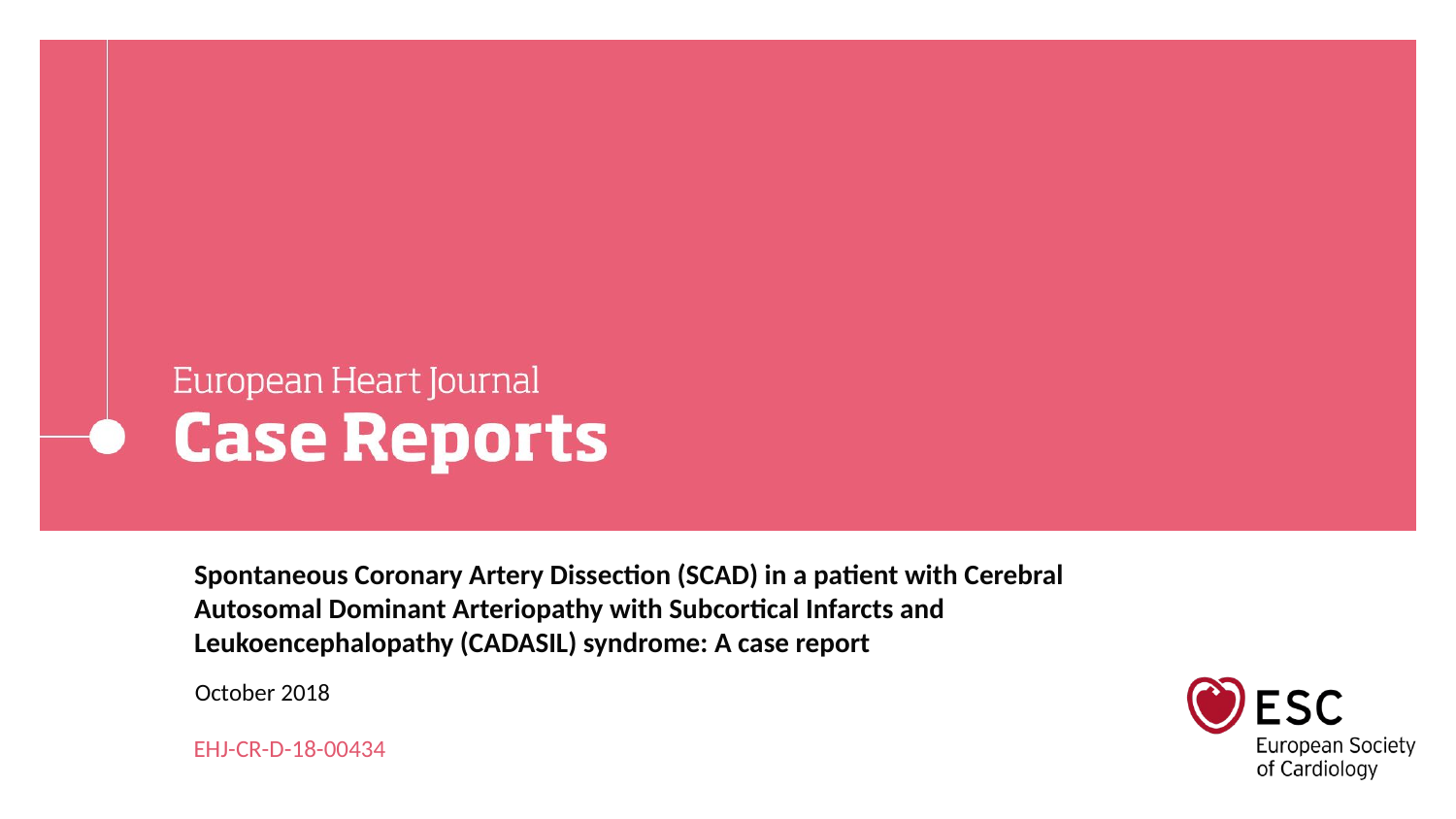

# Spontaneous Coronary Artery Dissection (SCAD) in a patient with Cerebral Autosomal Dominant Arteriopathy with Subcortical Infarcts and Leukoencephalopathy (CADASIL) syndrome: A case report
October 2018
EHJ-CR-D-18-00434

## Slide 2
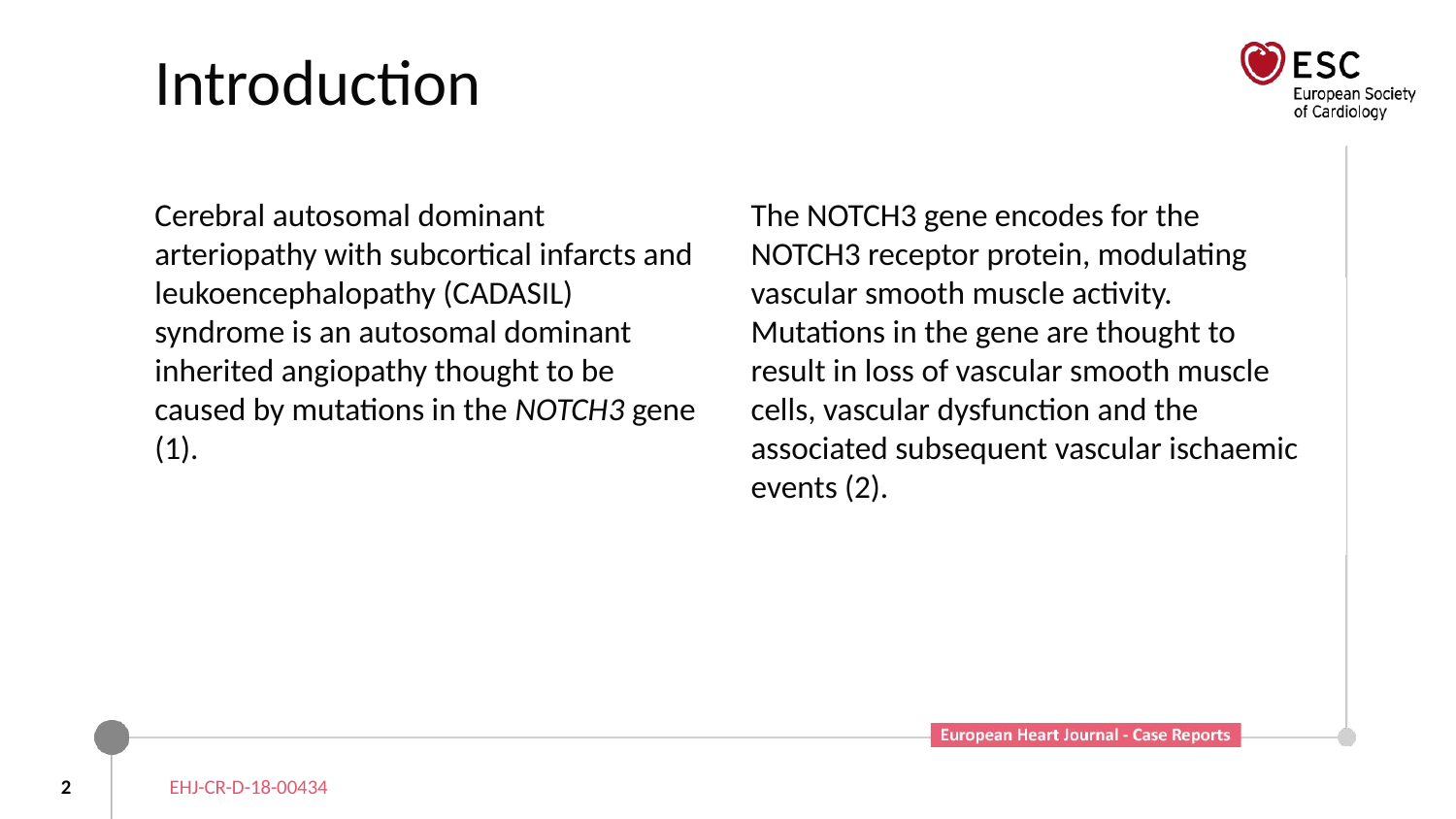

# Introduction
Cerebral autosomal dominant arteriopathy with subcortical infarcts and leukoencephalopathy (CADASIL) syndrome is an autosomal dominant inherited angiopathy thought to be caused by mutations in the NOTCH3 gene (1).
The NOTCH3 gene encodes for the NOTCH3 receptor protein, modulating vascular smooth muscle activity. Mutations in the gene are thought to result in loss of vascular smooth muscle cells, vascular dysfunction and the associated subsequent vascular ischaemic events (2).
2
EHJ-CR-D-18-00434

## Slide 3
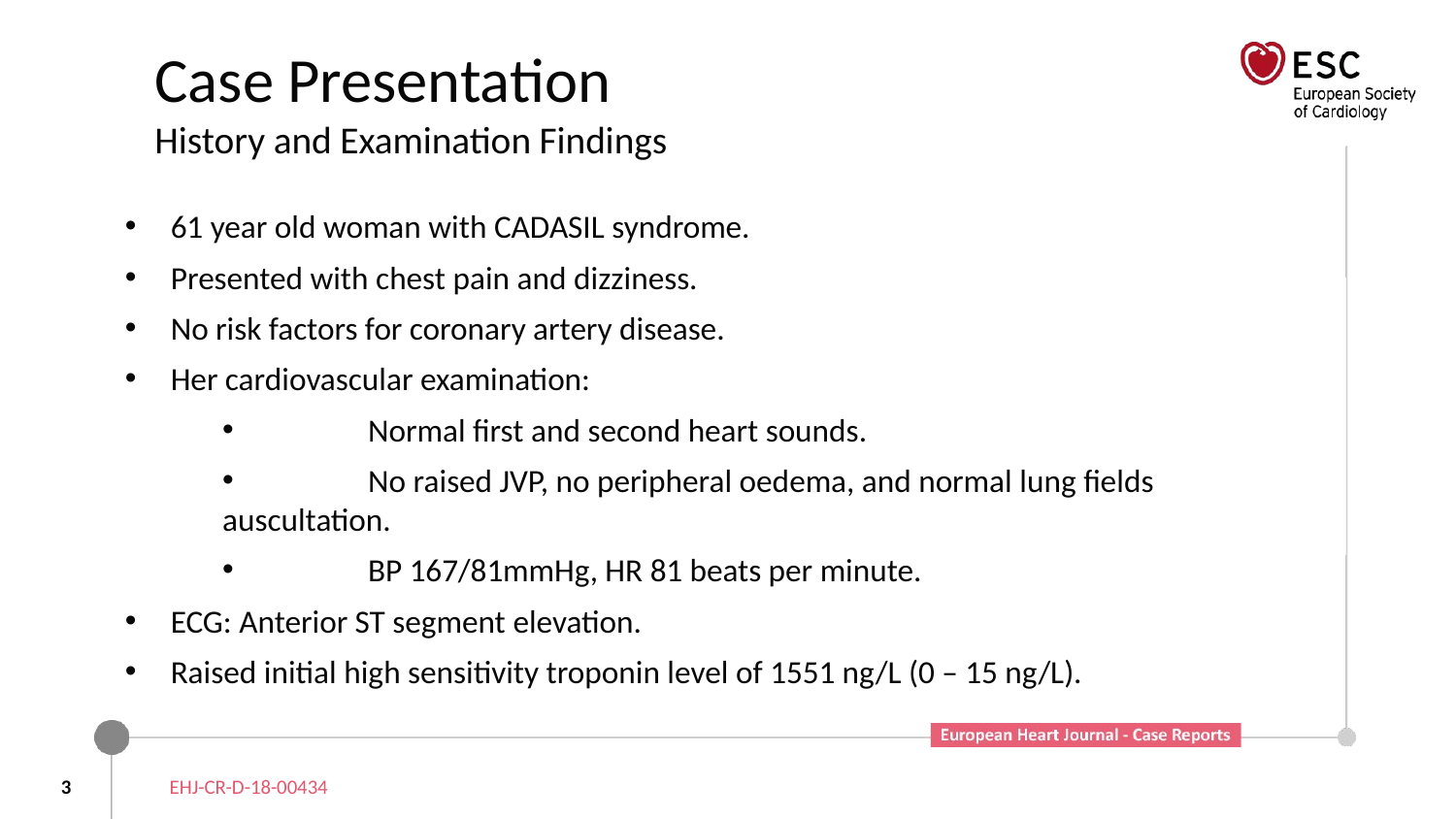

# Case Presentation History and Examination Findings
61 year old woman with CADASIL syndrome.
Presented with chest pain and dizziness.
No risk factors for coronary artery disease.
Her cardiovascular examination:
	Normal first and second heart sounds.
	No raised JVP, no peripheral oedema, and normal lung fields auscultation.
	BP 167/81mmHg, HR 81 beats per minute.
ECG: Anterior ST segment elevation.
Raised initial high sensitivity troponin level of 1551 ng/L (0 – 15 ng/L).
3
EHJ-CR-D-18-00434

## Slide 4
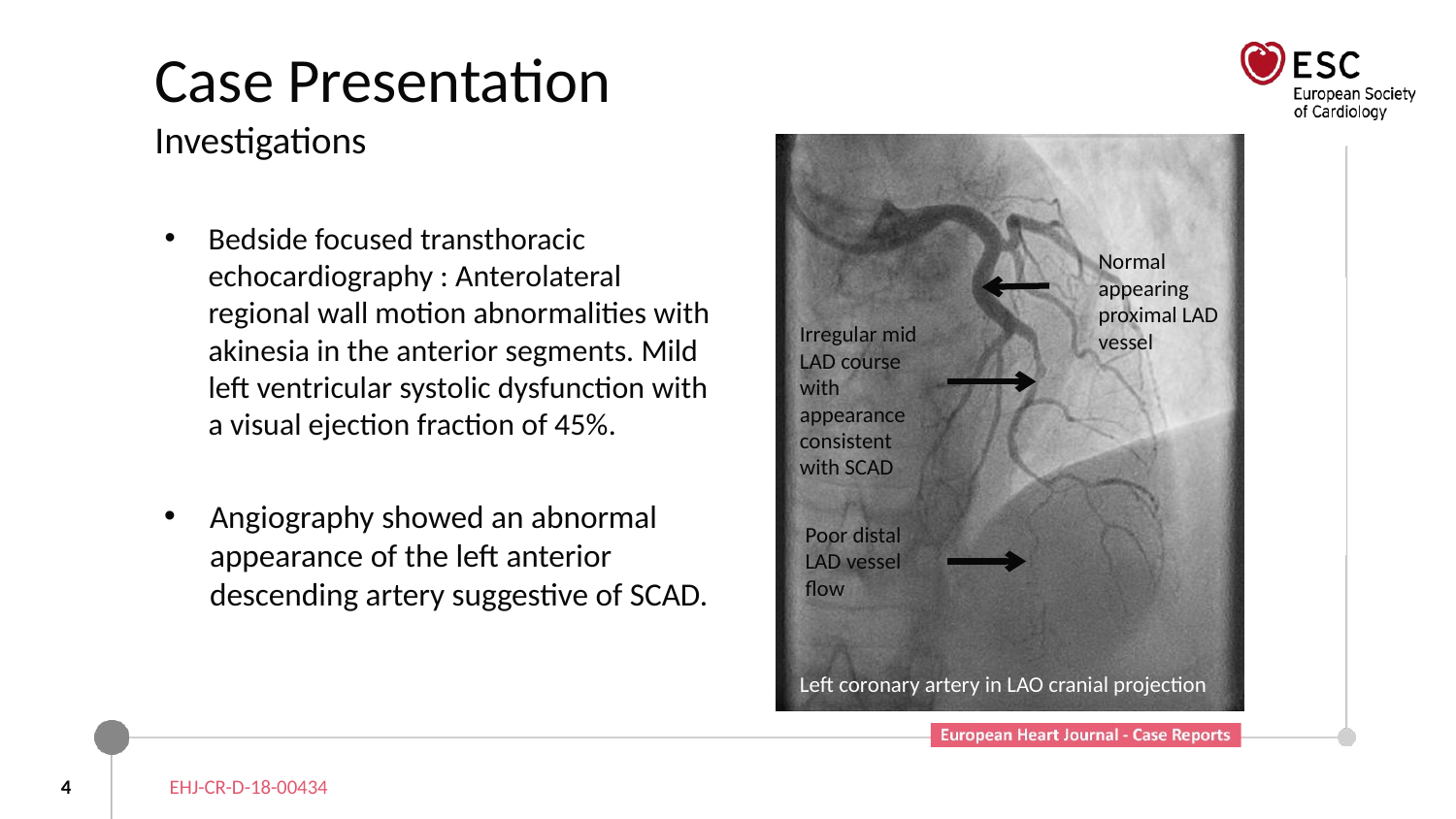

# Case Presentation Investigations
Bedside focused transthoracic echocardiography : Anterolateral regional wall motion abnormalities with akinesia in the anterior segments. Mild left ventricular systolic dysfunction with a visual ejection fraction of 45%.
Normal appearing proximal LAD vessel
Irregular mid LAD course with appearance consistent with SCAD
Angiography showed an abnormal appearance of the left anterior descending artery suggestive of SCAD.
Poor distal LAD vessel flow
Left coronary artery in LAO cranial projection
4
EHJ-CR-D-18-00434

## Slide 5
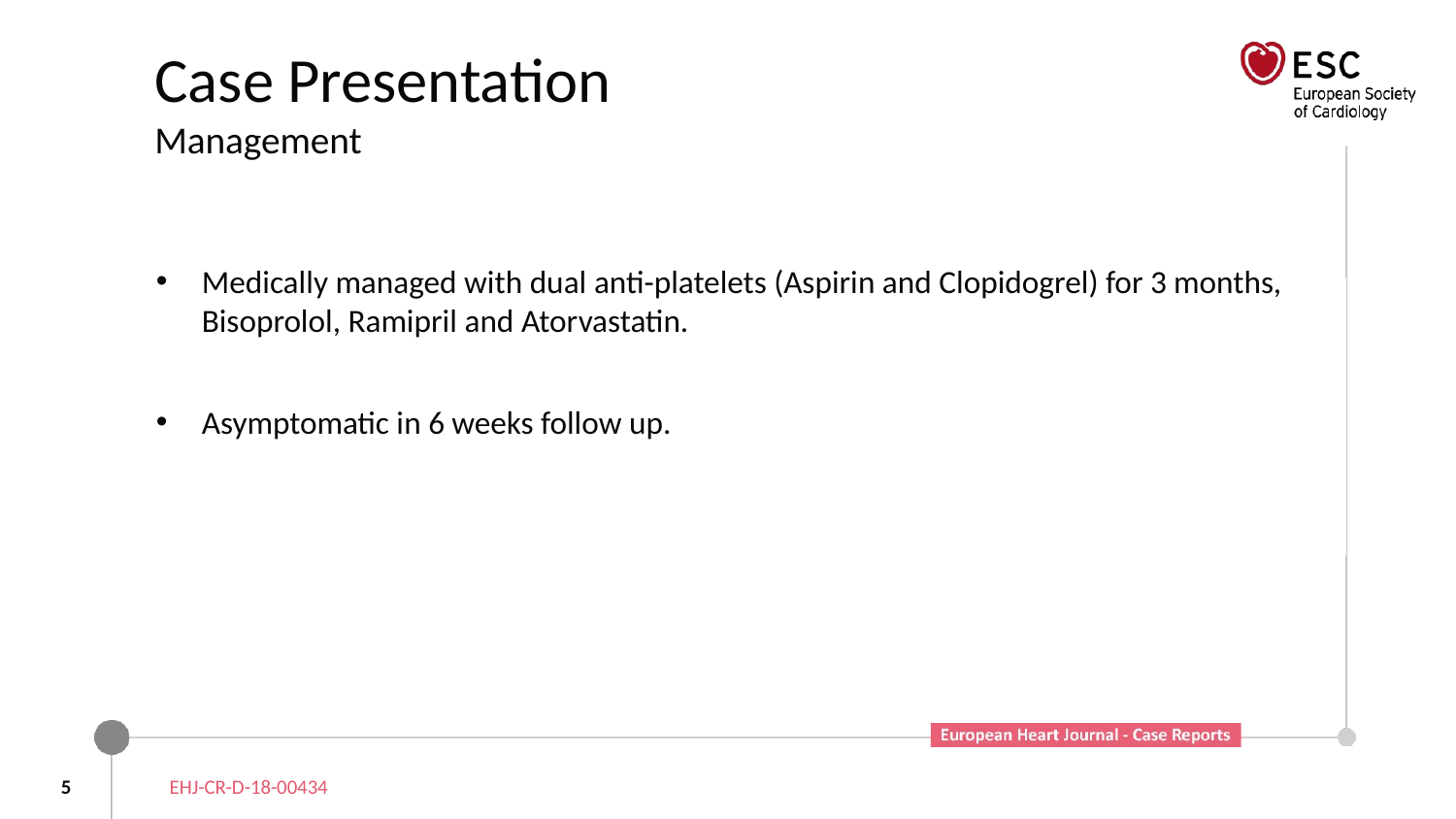

# Case Presentation Management
Medically managed with dual anti-platelets (Aspirin and Clopidogrel) for 3 months, Bisoprolol, Ramipril and Atorvastatin.
Asymptomatic in 6 weeks follow up.
5
EHJ-CR-D-18-00434

## Slide 6
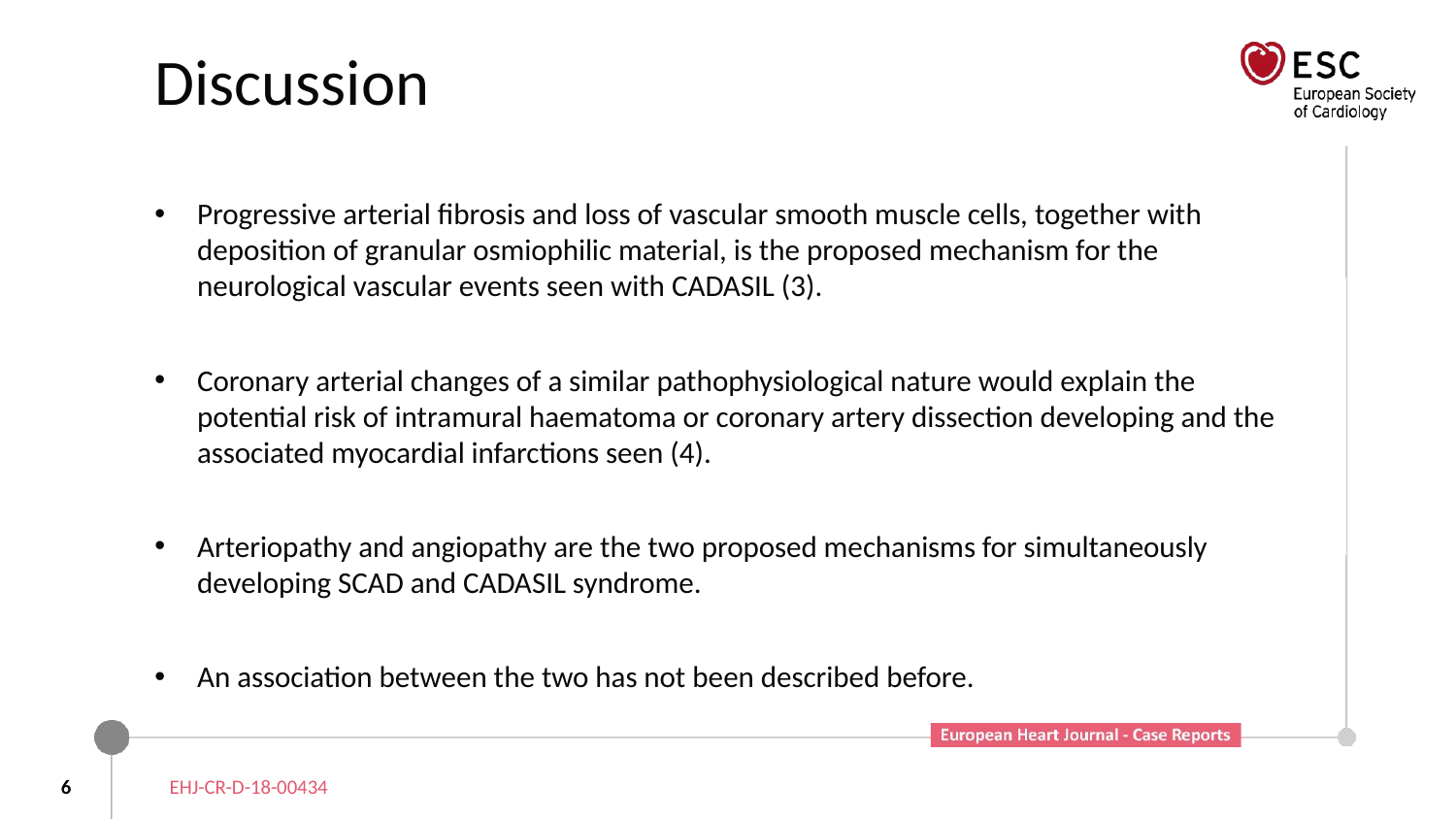

# Discussion
Progressive arterial fibrosis and loss of vascular smooth muscle cells, together with deposition of granular osmiophilic material, is the proposed mechanism for the neurological vascular events seen with CADASIL (3).
Coronary arterial changes of a similar pathophysiological nature would explain the potential risk of intramural haematoma or coronary artery dissection developing and the associated myocardial infarctions seen (4).
Arteriopathy and angiopathy are the two proposed mechanisms for simultaneously developing SCAD and CADASIL syndrome.
An association between the two has not been described before.
6
EHJ-CR-D-18-00434

## Slide 7
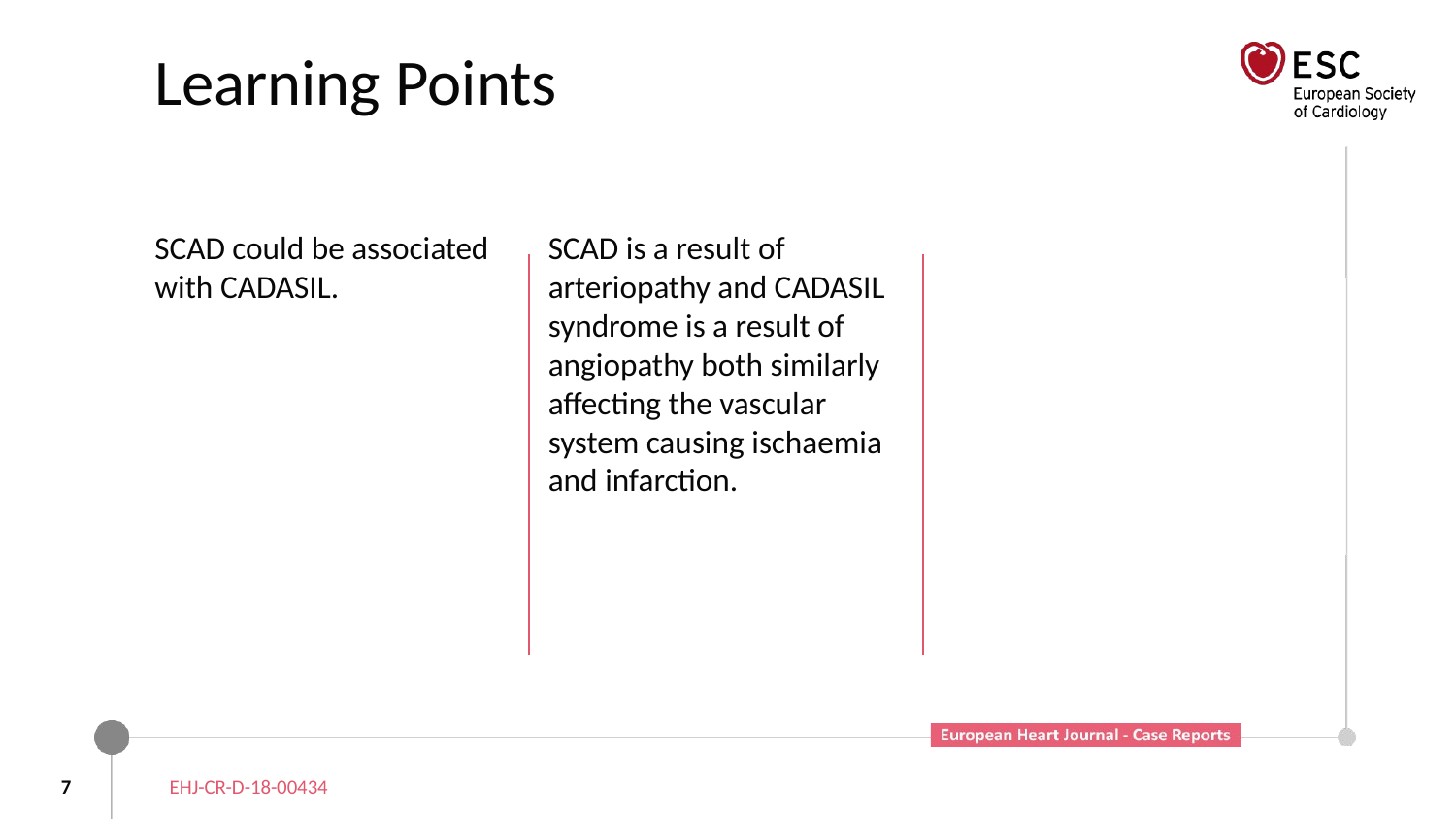

# Learning Points
SCAD could be associated with CADASIL.
SCAD is a result of arteriopathy and CADASIL syndrome is a result of angiopathy both similarly affecting the vascular system causing ischaemia and infarction.
7
EHJ-CR-D-18-00434

## Slide 8
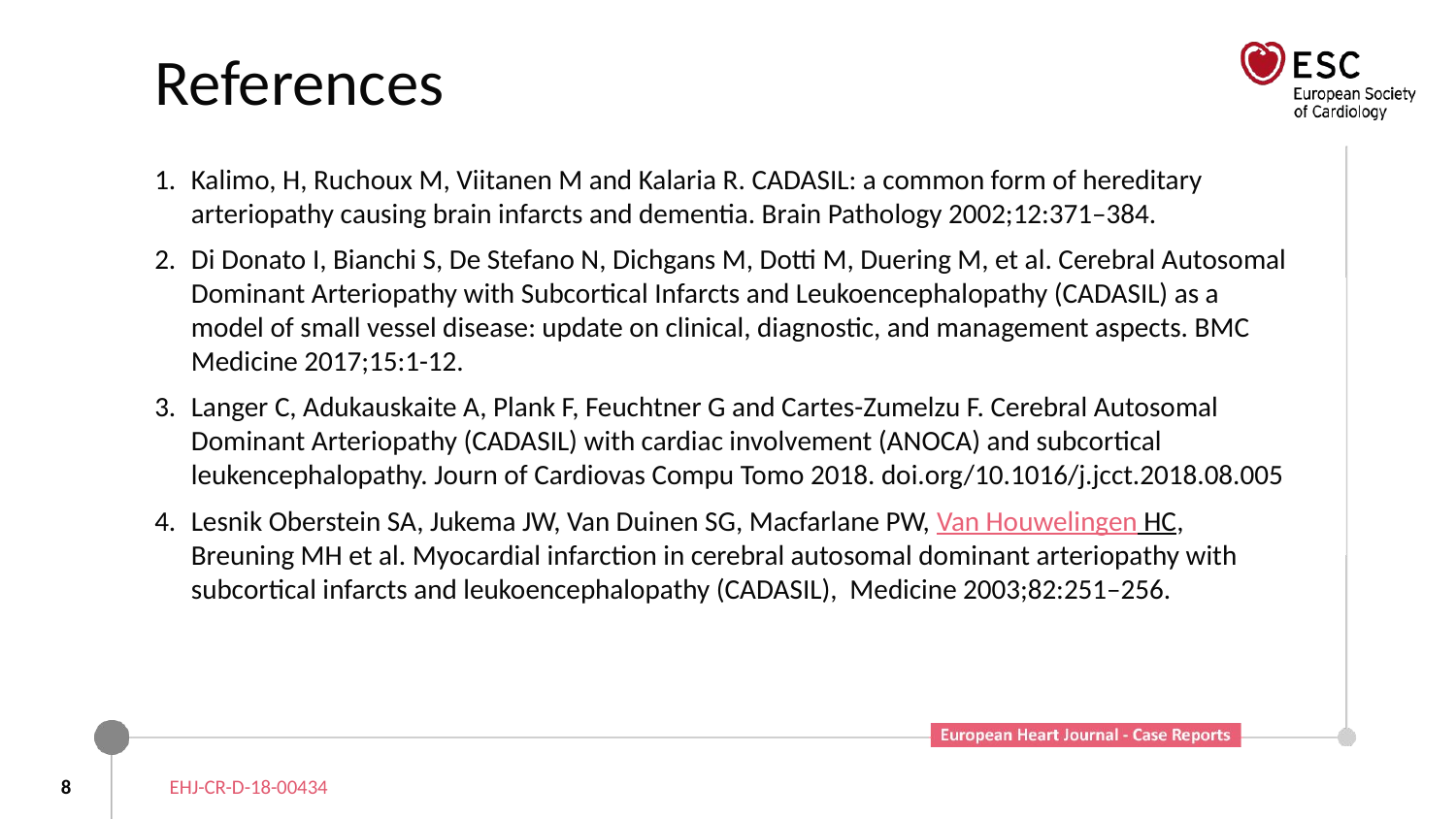

# References
Kalimo, H, Ruchoux M, Viitanen M and Kalaria R. CADASIL: a common form of hereditary arteriopathy causing brain infarcts and dementia. Brain Pathology 2002;12:371–384.
Di Donato I, Bianchi S, De Stefano N, Dichgans M, Dotti M, Duering M, et al. Cerebral Autosomal Dominant Arteriopathy with Subcortical Infarcts and Leukoencephalopathy (CADASIL) as a model of small vessel disease: update on clinical, diagnostic, and management aspects. BMC Medicine 2017;15:1-12.
Langer C, Adukauskaite A, Plank F, Feuchtner G and Cartes-Zumelzu F. Cerebral Autosomal Dominant Arteriopathy (CADASIL) with cardiac involvement (ANOCA) and subcortical leukencephalopathy. Journ of Cardiovas Compu Tomo 2018. doi.org/10.1016/j.jcct.2018.08.005
Lesnik Oberstein SA, Jukema JW, Van Duinen SG, Macfarlane PW, Van Houwelingen HC, Breuning MH et al. Myocardial infarction in cerebral autosomal dominant arteriopathy with subcortical infarcts and leukoencephalopathy (CADASIL), Medicine 2003;82:251–256.
8
EHJ-CR-D-18-00434
